# Optimization of macronutrients for improved grain yield of quinoa (*Chenopodium quinoa* Wild.) crop under semi-arid conditions of Morocco

**DOI:** 10.3389/fpls.2023.1146658

**Published:** 2023-06-27

**Authors:** Nawal Taaime, Khalil El Mejahed, Redouane Choukr-Allah, Rachid Bouabid, Abdallah Oukarroum, Mohamed El Gharous

**Affiliations:** ^1^ Agricultural Innovation and Technology Transfer Center, Agrobiosciences, Mohammed VI Polytechnic University, Ben Guerir, Morocco; ^2^ Department of Agronomy, National School of Agriculture, Meknes, Morocco; ^3^ Plant Stress Physiology Laboratory, Agrobiosciences, Mohammed VI Polytechnic University, Ben Guerir, Morocco

**Keywords:** quinoa, nitrogen, phosphorus, potassium, fertilization, semi-arid, Morocco

## Abstract

In the context of climate change, quinoa represents a potential alternative crop for increasing crops diversity, agricultural productivity, and farmer’s income in semi-arid regions. However, appropriate crop management practices under limited water supply are still poorly documented. Quinoa, like other cultivated crops, needs optimum quantities of nutrients, especially nitrogen (N), phosphorus (P), and potassium (K), for better growth and high grain yield. To determine the adequate levels of nutrient requirements and their effect on quinoa growth and productivity, a field experiment was conducted during two growing seasons (2020–2021 and 2021–2022). The experiment was conducted in Ben Guerir region, north-central Morocco, and consisted of a randomized complete block design (RCBD) with three replications. The treatments studied consist of a combination of four N rates (0, 40, 80, and 120 kg ha^−1^), three P rates (0, 30, and 60 kg P_2_O_5_ ha^−1^), and three K rates (0, 60, and 120 kg K_2_O ha^−1^). The physiological, nutritional, and production parameters of quinoa were collected and analyzed. The results showed that the highest total biomass (3.9 t ha^−1^) and grain yield (0.8 t ha^−1^) under semi-arid conditions were obtained with 40 kg N ha^−1^, 60 kg P_2_O_5_ ha^−1^, and 120 kg K_2_O ha^−1^. The application of 40–60–120 kg ha^−1^ of N–P_2_O_5_–K_2_O increased plant height by 44%, chlorophyll content index by 96%, total biomass by 134%, grain yield by 112%, and seed weight by 118%. Among the three macronutrients, N was the most limiting factor, followed by K and P. Nutrients uptake data showed that quinoa needs 60 kg N, 26 kg P_2_O_5_, and 205 kg K_2_O to produce 1 t of grain yield. Our field results provide future recommendations for improving the agronomic and environmental sustainability of quinoa cultivation in dryland areas in Morocco.

## 1 Introduction

Quinoa (*Chenopodium quinoa* Willd.) originated from the Andean region of South America (Adolf et al., 2013). This crop has received much attention because of its high nutritional value and its high tolerance to frost, drought, and salinity (Jensen et al., 2000; Jacobsen, 2003; Jacobsen et al., 2005; Adolf et al., 2013). Quinoa is considered a potential novel crop in other locations across the globe because of its high adaptation to climate change and its high economic value (Bazile et al., 2016).

Quinoa was introduced in Morocco in 1999 (Benlhabib, 2005). Nevertheless, much information is needed about its production techniques and its response to different environments for better adaptation under dryland farming in arid and semi-arid areas of Morocco. Quinoa is well adapted to marginal soils (Choukr-Allah et al., 2016). However, nutrients deficiency limits crop production (Aquino et al., 2013). Determining quinoa macronutrient requirements is crucial to maintain the crop metabolism for optimal growth and development (Sales et al., 2021). Unlike the cultivation of major crops, literature reference about quinoa nutrient needs and management in arid regions is still lacking.

Plants need nitrogen (N) for optimal growth and development (Sales et al., 2021). Because of its structural role in chlorophyll and in nucleic and amino acids composition, insufficient quantities of N result in very slow growth, stunted plant, and light green to yellow foliage color (Benton Jones, 2012). N application has been reported to enhance crop growth, productivity, and quality. Moreover, it was found that N improves quinoa drought tolerance and improves seed yield and quality (Alandia et al., 2016). Studies evaluating the N effect on quinoa recorded a positive response to N application up to 120 kg N ha^−1^, with 96% yield increase compared to the control (Schulte auf’m Erley et al., 2005). However, the yield increase was smaller when N application rate increased from 120 to 160 kg N ha^−1^, achieving only 2.7% (Jacobsen et al., 1994). When quinoa was cultivated with drip irrigation under arid conditions, 90 kg N ha^−1^ was recommended for optimum yields (4.5 t ha^−1^), while higher rates favored vegetative growth and increased the incidence of mildew (Quispe, 2018). Quinoa seemed to respond to high N levels up to 225 kg ha^−1^, with a grain yield of 2.3 and 3.6 t ha^−1^ for two cultivars Faro and UDEC10, respectively (Berti et al., 2000). Shams (2012) also evaluated the response of quinoa to higher N rates in sandy soils. He found that the maximum economic yield, 1.1 t ha^−1^, was achieved with 360 kg N ha^−1^, being almost 11 times over control.

Unlike the N, studies on phosphorus (P) nutrition of quinoa are still scarce. Optimal levels of P enhance the growth and productivity in a sustainable agricultural system (Schröder et al., 2011). However, adequate quantities of this nutrient for optimal growth and productivity of quinoa under semi-arid conditions are still lacking. Thus, evaluating P response and optimizing P application rate will provide future recommendations for improving the agronomic and environmental sustainability of quinoa cultivation in dryland areas. Under P-deficient conditions, quinoa plants display necrosis of the lower leaves, and the upper leaves become pale green (Sales et al., 2021). This deficiency affects the photosynthetic activity due to the negative effect on ATP synthesis and Rubisco regeneration, which limit the plant metabolism, the formation of the phospholipids, and, consequently, new cells for the growth and development of quinoa plants (Sales et al., 2021). P also promotes root growth and activity, increases the area of contact between the root and soil, and therefore enhances the drought resistance of quinoa (Pang et al., 2017). The application of P has been reported to enhance the growth and development of quinoa. Llaca Ninaja (2014) studied the effect of four P rates of 0, 40, 80, and 120 kg P_2_O_5_ ha^−1^ and found that 88 kg P_2_O_5_ ha^−1^ was needed for an optimal yield of 2.9 t ha^−1^. Higher P rates resulted in lower yields. In another study (Quispe, 2018), increasing P rate from 90 to 180 kg ha^−1^ did not significantly affect the growth characteristics and yield of quinoa, with an average of 4.1 t ha^−1^. Likewise, Tapia (1997) recommended the application of 60–80 kg ha^−1^ of P when annual precipitations exceeded 600 mm. Under saline conditions, P application at 60 and 70 kg P_2_O_5_ ha^−1^ has been found to minimize the salt stress effect and increase the yield by 29% and 51% at low salinity level (5 dS m^−1^) and by 13% and 8% at high salinity level (12 dS m^−1^) (Bouras et al., 2021).

In addition, K is an important nutrient that ensures plant growth even under abiotic stress (Turcios et al., 2021). The response of quinoa to K fertilization is still misunderstood. This is due to the great availability of this element in its area of cultivation (Mujica et al., 2001). Thus, research studying the effect of K on quinoa crop are scarce. According to Mujica et al. (2001), quinoa is very demanding in K. When available in insufficient quantities, chlorosis appears at the margins of older leaves, followed by necrosis (Sales et al., 2021). In Central Highlands of Vietnam, application of 105 kg K_2_O ha^−1^ was found to be the optimum rate of K for quinoa production in ferralsols and acrisols (Minh et al., 2022).

Plants’ nutrient requirements differ according to the crop, cultivar, potential yield, soil composition, and environmental conditions (Cottenie, 1980; Shand et al., 2006). Under water-limited conditions, quinoa nutrient requirements are supposed to be lower than optimal conditions. To our knowledge, no work has attempted to optimize macronutrient fertilization for maximum yield of quinoa in semi-arid regions of Morocco, and literature on combined application of N, P, K, and their uptake by quinoa crop in semi-arid regions of Morocco is still limited. We hypothesize that N, P, and K fertilization enhances quinoa physiological, growth, and productivity parameters. To test this hypothesis, we conducted the present study with the aim to (1) evaluate the effect of macronutrient on quinoa morphological and physiological parameters; (2) optimize N, P, and K application for high productivity and nutrient use efficiency; and (3) study the effect of macronutrients on quinoa nutritional status and determine N, P, and K uptake for maximum quinoa grain yield under semi-arid conditions.

## 2 Materials and methods

### 2.1 Experimental site

The field experiment was conducted at the experimental farm of Mohamed VI Polytechnic University in Benguerir (32°13.0800 N, 7°53.230 W), Morocco. This region is characterized by an arid climate with an average annual precipitation of 190 mm and an average annual temperature of 19.5°C (40 years of data) (Taaime et al., 2022b). The experiment was conducted during two cropping seasons 2020–2021 and 2021–2022, between December and May.

A composite soil sampling, with 18 soil subsamples collected from the 20 cm topsoil, was analyzed for chemical properties. The soil was dried, crushed, and sieved to 2 mm. The pH and electrical conductivity (ECe) were measured in a 1:5 soil:water extract. Soil organic matter (OM) was determined using sulfochromic oxidation of carbon in a mixture of potassium dichromate and sulfuric acid at 135°C according to Walkley and Black (1934). P was measured using the Olsen method (Olsen et al., 1954) and calcium carbonate using chlorohydric acid (Allison, 1960). Sodium (Na), K, and magnesium (Mg) were extracted by the ammonium acetate at pH = 7 and determined by atomic absorption spectroscopy (Agilent Technologies. 200 Series AA). Calcium oxide was measured using hydrochloric acid (Sparks et al., 1996), and total N was determined using the Kjeldahl method (Weaver et al., 1994). Nitrate and ammoniacal N were measured by extraction with KCl using a continuous flow analyzer (Skalar Analytical), and zinc (Zn), iron (Fe), manganese (Mn), and copper (Cu) were determined using the DTPA extraction at pH = 7.3 (Lindsay and Norvell, 1978).

The result showed that the soil has a loamy texture, with 1.86% of organic matter, a pH of 8.1, and an EC_e_ of 0.19 dS m^−1^. During both growing seasons, the soil has a high content of K and P. Other chemical parameters of the soils are presented in **Table 1**.

**Table 1:** Chemical characteristic of the experiment soils during the 2020–2021 and 2021–2022 cropping seasons.

	CaCO_3_	Total N	${\text{NH}}_{4}^{+}$	${\text{NO}}_{3}^{-}$	P_2_O_5_	K_2_O	Na_2_O	MgO	CaO	Cu	Mn	Fe	Zn	CEC
	%		mg kg^−1^											meq/100 g
2020–2021	0.1	0.11	2.4	43.2	40	456	135	750	5004	0.7	5.1	4.6	0.4	17.2
2021–2022	0.1	0.12	5.2	72.2	47	377	679	711	6870	0.9	7.5	3.4	0.4	17.6

### 2.2 Experimental set-up

To determine the optimal fertilizers rate for quinoa growth and productivity, four nitrogen rates (0, 40, 80, and 120 kg N ha^−1^) combined with three P rates (0, 30, and 60 kg P_2_O_5_ ha^−1^) and three K rates (0, 60, and 120 kg K_2_O ha^−1^) were used. For each treatment, half of the amount of N was applied before sowing as ammonium sulfate, and the other half was applied 50 days after sowing as ammonium nitrate, as recommended by Spehar et al. (2015). The total amounts of P and K were applied before planting as triple superphosphate and potassium sulfate, respectively. The experiment layout was a randomized complete block design (RCBD) with three replicates. The experimental plots were 7.5 m long and 3.5 m wide (seven rows at a 0.5 m inter-row distance). The quinoa genotype used was ICBA-Q5 because of its adaptation to arid Moroccan conditions (Hirich et al., 2021).

### 2.3 Agronomic practices

Chisel plow followed by disk harrow were used for seedbed preparation. Quinoa was cultivated in virgin soil during 2020–2021 and after the quinoa crop during 2021–2022 growing season. Quinoa was sown mechanically with a seed drill (Sembradoras GIL, SAX-19-M), at the rate of 8 kg ha^−1^ and tinned at the ramification stage to 20–25 plants per m^2^. Weeding was done manually 1 month after the planting, and fungicide application (3.88% metalaxyl-M + 64% mancozeb) was done at a rate of 500 g ha^−1^ to control the mildew.

Supplemental irrigation (SI) was applied by a drip irrigation system during the early vegetative growth, flowering, and seed-filling stages to ensure grain production because drought occurred during the experiment years. The drip lines were spaced by 50 cm, with 1.2 L h^−1^ integral drippers spaced 30 cm apart. During the prementioned development stages, daily water volumes were estimated using the standard formula: $SI=\frac{\left(Kc*ET0\right)}{2}$ , where Kc is the quinoa crop coefficient factor being 0.5 at plant establishment and 1 during flowering and seed filling (Garcia et al., 2003), ET_0_ is the reference evapotranspiration obtained from the weather station at the UM6P experimental farm, and “e” is the irrigation system efficiency being equal to 80%. The total amount of irrigation water was estimated to be 129 and 122 mm during the first and second cropping seasons, respectively. We represent in **Table 2** the characteristics of the irrigation water applied during the plant establishment, flowering, and seed filling.

**Table 2:** Characteristics of irrigation water of the experiment.

Parameter	pH	E_C_ (mS cm^−1^)	P_2_O_4_ (mg l^−1^)	NO_3_ (mg l^−1^)	NH_4_ (mg l^−1^)	K (mg l^−1^)	Na (mg l^−1^)	Ca (mg l^−1^)	Mg (mg l^−1^)	Cl (mg l^−1^)	SO_4_ (mg l^−1^)
Values	8.5	3.3	6.98	22.0	14.8	34.5	378.8	80.6	66.2	529.7	199.9

### 2.4 Growth and yield parameters measured

#### 2.4.1 Plant height

Before the flowering stage, 10 plants were randomly selected from each plot after eliminating the borders from measurements, and the plant height (cm) was measured from the ground level to the top of quinoa panicle of the mean stem (Hussain et al., 2020).

#### 2.4.2 Chlorophyll content index

Similarly, 10 quinoa plants were randomly selected in each experimental unit before the flowering stage. The CCI was measured for the two youngest fully expanded leaves of the main stem using a chlorophyllometer (Opti-Sciences, CCM-200) (Amjad et al., 2015).

#### 2.4.3 Grain yield and its components

In each experimental unit, three quadrates of 1 m^2^ were harvested, air-dried, and weighted to determine the total biomass (g m^−2^). Afterward, quinoa plants were threshed, and grains were separated from the straw part and weighed to determine the grain yield (g m^−2^). Harvest Index (HI; %) was calculated as the ratio between the grain yield and total above ground biomass. Thousand seed weight (TSW; g) was measured for each treatment.

### 2.5 Nutrient use efficiency

N use efficiency (NUE; kg/kg) is defined as the amount of grain produced for each kilogram of N applied. The same definition was followed for P use efficiency (PUE) and K use efficiency (KUE). NUE was measured following Belete et al. (2018), using the formula:


$$\begin{array}{c} \text{NUE}\left(\text{kg/kg}\right)\\ =\frac{Grain\;yield\;in\;the\;fertilized\;plots\;\left(kg\right)-Grain\;yield\;in\;the\;unfertilized\;plot\;\left(kg\right)}{Quantity\;of\;fertilizer\;applied\;\left(kg\right)} \end{array}$$

### 2.6 Nutrient uptake

Total plant biomass at physiological maturity was used to determine the N, P, and K content. Samples were ground to pass through a screen with 225-µm openings. Two grams of the ground material, for each treatment’s replication, was digested with salicylic acid and sulfuric acid, and N content was determined colorimetrically on a Skalar San++. Concerning P and K contents, 0.5 g of the ground material was digested with nitric acid. Then, the digested material was analyzed, after filtration, for P and K using inductively coupled plasma–optical emission spectrometry (ICP-OES).

### 2.7 Statistical analysis

Quinoa height, CCI, total biomass, grain yield, TSW, and nutrient uptake and use efficiency were used to evaluate the effect of N, P, and K rates and their interaction using three-way ANOVA test. When ANOVA test was significant, means were compared using Student–Newman–Keuls (S–N–K) test, and all statistical differences were tested at 5% probability level or lower (α ≤ 0.05). Statistical analyses were performed using the SPSS program (Version 20, IBM SPSS Inc., Chicago, IL, USA).

## 3 Results

### 3.1 Climatic conditions of the experimental site

**Figure 1:**
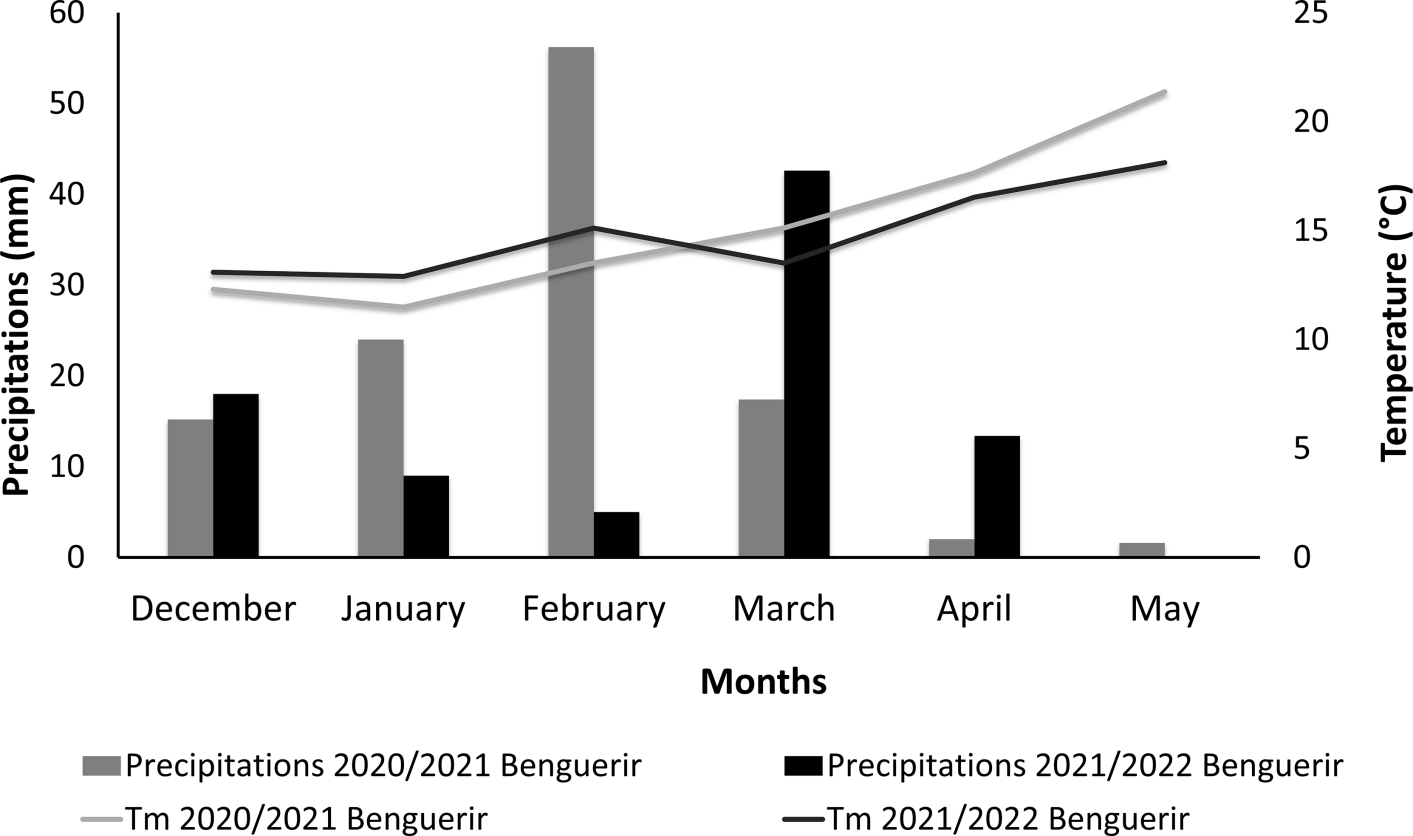
Precipitations and average temperature during the two growing seasons of quinoa.

The experiment was conducted under Mediterranean arid climate. The winter season goes from November to March. The crop received 116 and 88 mm of rain during 2020–2021 and 2021–2022 cropping seasons, respectively (**Figure 1**). The maximum amount of rain received was 56 and 43 mm in February 2021 and March 2022, respectively. The average temperature ranged from 13.1°C to 21.4°C during 2020–2021 and from 12.9°C to 18.1°C during 2021–2022 cropping season. The first year of the experiment (2020–2021) was characterized by high temperatures at the end of the growing cycle. The absolute maximum temperature was 46°C (data not presented in the graph) and was recorded in May 2021. The total amount of water received by the crop (rainwater + irrigation) was estimated to be 245 and 210 mm during 2020–2021 and 2021–2022 cropping seasons, respectively.

### 3.2 Effect of fertilization on plant height

**Figure 2:**
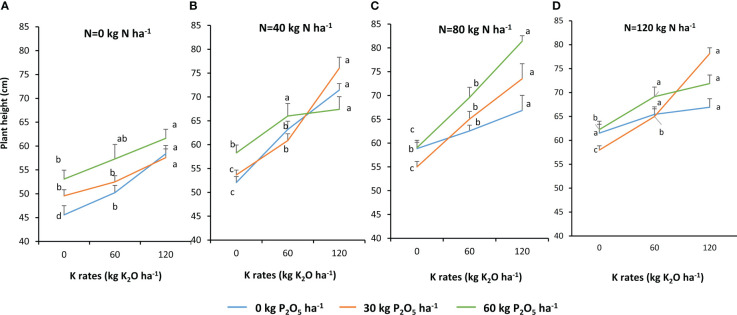
Plant height in response to N, P and fertilizers (**A**. N=0 kg N/ha, **B**. N=40 kg/ha, **C**. N=80 kg/ha, and **D**. N=120 kg/ha). Data are presented by the mean of the two growing seasons 2020-2021 and 2021-2022. For each P rate, means followed by the same letters are not significantly different at p ≤0.05.

There was a significant combined effect of N, P, and K fertilization on quinoa plant height (**Figure 2**). This parameter increased from 45.6 cm with no fertilizer application to 81.4 cm with 80–60–120 kg ha^−1^ of N–P_2_O_5_–K_2_O, with a 79% increase over the control. Individual effects of N, P, and K fertilization were also observed. Among the three nutrients, N had the highest effect on quinoa plant height. This parameter increased from 45.57 cm with no fertilizer application to 61.52 cm with the application of 120 kg N ha^−1^, being 35% over the control. K and P had lower effect on quinoa plant height. K at 120 kg K_2_O ha^−1^ and P at 60 kg P_2_O_5_ ha^−1^ increased plant height by 28% and 17%, respectively.

### 3.3 Effect of fertilization on CCI

**Figure 3:**
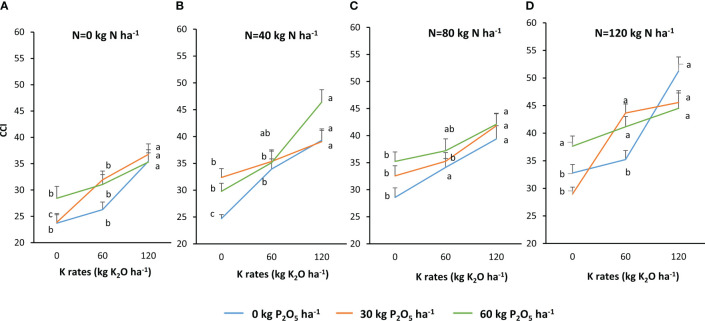
Chlorophyll content index (CCI) in response to N. P and K fertilizers. (**A**. =0 kg N/ha, **B**. =40 kg/ha, **C**. =80 kg/ha, and **D**. =120 kg/ha) Data are presented by the mean of the two growing seasons 2020-2021 and 2021-2022. For each P rate, means followed by the same letters are not significantly different at p ≤0.05.

The statistical analysis of the plant CCI showed that there was an interaction between N, P, and K fertilization (**Figure 3**). Application of 40–60–120 kg of N–P_2_O_5_–K_2_O were the optimal N, P, and K rates for increasing the CCI, with 94% higher than the control. Similarly to the plant height, the individual effect of N, P, and K fertilization was recorded. Marginal means showed that the plant CCI increased by 32% from 0 to 120 kg N ha^−1^. P has a lower effect than N on plant CCI. The application of 60 kg P_2_O_5_ ha^−1^ increased this parameter by 10% compared to the control. Concerning the K, the plant CCI recorded a high response to the application of this nutrient, with the maximum increase (38%) observed at 120 kg K_2_O ha^−1^.

### 3.4 Effect of fertilization on total biomass

**Figure 4:**
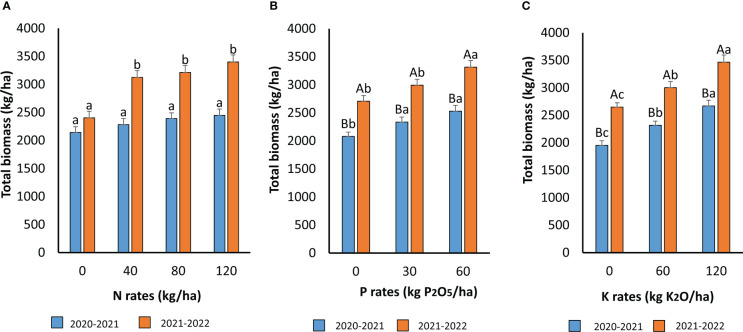
Plant total biomass as affected by N **(A)**, P **(B)** and K **(C)** application during the two cropping seasons 2020-2021 and 2021-2022. For **(A)** means followed by the same small letters are not significantly different at p ≤0.05. For **(B, C)** for each P and Krate, means followed by the same capital letters are not significantly different at p ≤0.05. For each growing season, means followed by the same small letters are not significantly different at p ≤0.05.

The effect of N, P, and K fertilizers on quinoa total biomass is presented in **Figure 4**. No interaction between the three nutrients on total plant biomass was recorded. However, the effect of the growing season was highly significant. The response of total biomass to N application differed between 2020–2021 and 2021–2022 cropping seasons. The first growing season (2020–2021) was characterized by temperatures higher than the second growing season (2021–2022). The addition of N during 2020–2021 slightly increased the plant total biomass, but the difference between N rates was not significant (**Figure 4A**). However, during the second growing season, when the climate was cooler, the application of N significantly increased total plant biomass. This parameter increased by 29% from 2.4 t ha^−1^ with no N application to 3.4 t ha^−1^ with 120 kg N ha^−1^. However, it was observed that the optimal N rate was 40 N kg ha^−1^, since no significant difference was recorded with higher N application rates.

The application of P significantly enhanced the plant’s total biomass (**Figure 4B**). The effect of the growing season was also significant, and higher total biomass was recorded during the second growing season. The optimal rate was 60 kg P_2_O_5_ ha^−1^, resulting in the highest plant biomass of 2.5 and 3.3 t ha^−1^ during 2020–2021 and 2021–2022 growing seasons, respectively. The P at the rate of 60 kg P_2_O_5_ ha^−1^ increased the plant’s total biomass by 18% in both seasons. K application and growing season also significantly affected the total crop biomass (**Figure 4C**). The application of 120 kg K_2_O ha^−1^ resulted in the highest plant biomass, 2.7 and 3.5 t ha^−1^, during 2020–2021 and 2021–2022 growing seasons, respectively. When comparing the three nutrients, N application has the highest effect on plant total biomass, followed by K and P applications.

### 3.5 Effect of fertilization on grain yield

**Figure 5:**
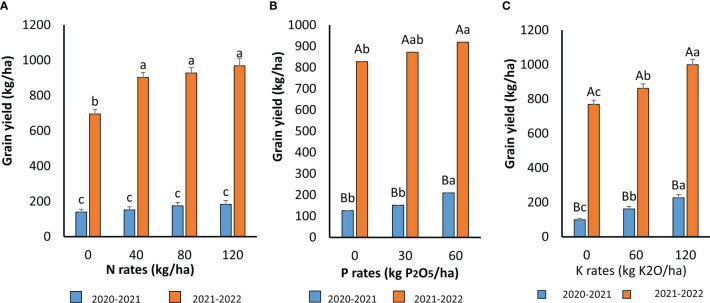
Grain yield as affected by N **(A)**, P **(B)** and K **(C)** application during the two cropping seasons 2020- 2021 and 2021-2022. For **(A)** means followed by the same small letters are not significantly different at p 30.05 for **(B, C)** for each P and K rate, means followed by the same capital letters are not significantly different at p ≤0.05 For each growing season, means followed by the same small letters are not significantly different at p s0.05.

The effect of N, P, and K rates on quinoa grain yield is presented in **Figure 5**. The grain yield of quinoa followed the same trends as total biomass. There was no interaction between N, P, and K on quinoa grain yield. However, the growing season highly affected this parameter. There was a significant interaction between N application and the growing season. N did not significantly affect the quinoa grain yield during the first growing season (**Figure 5A**), with a slight increase from 0 to 120 kg N ha^−1^. This effect was significant during the second growing season, and the optimal N rate was 40 kg N ha^−1^, with a grain yield increase of 30% compared to the control. The high temperatures (up to 46°C) that occurred at the end of the first growing season affected the pollination and grain filling of quinoa, which was translated in low yields. The application of P was significant for both growing seasons (**Figure 5B**). The P applied at 60 kg P_2_O_5_ ha^−1^ was the optimal level to attain the highest quinoa grain yield with an increase of 16% compared to the control. A high difference between the grain yields of 2020–2021 and 2021–2022 growing seasons was recorded. High temperatures during the first growing season negatively affected the quinoa flowering and seed filling, which resulted in low grain yields. For the K application, quinoa highly responded to this nutrient addition. The amount of 120 kg K_2_O ha^−1^ increased the grain yield in both cropping seasons, being 25% higher than the control. Similarly to the other nutrients, grain yield differed highly between the two growing seasons. Again, the application of N resulted in the highest grain yield increase, followed by the K and P applications.

### 3.6 Effect of fertilization on harvest index and seed weight

**Figure 6:**
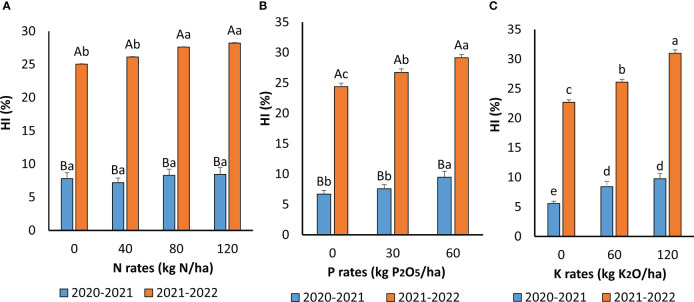
Harvest Index as affected by N **(A)**, P **(B)** and K **(C)** application during the two cropping seasons 2020-2021 and 2021-2022. For **(A, B)** means followed by the same small letters are not significantly different at p ≤0.05 For **(C)** for each N rate, means followed by the same capital letters are not significantly different at p ≤0.05. For each growing season, means followed by the same small letters are not significantly different at p ≤0.05.

The HI was significantly affected by N, P, and K applications (**Figure 6**). However, no interaction between these nutrients was recorded. N effect significantly differed between the growing seasons. During the first growing season (2020-2021), the application of N enhanced the HI but not significantly. Higher HI values were recorded during the second cropping season (2021–2022) and the application of 120 kg N ha^−1^ resulted in the highest value (28%). P and K addition significantly enhanced the HI. The effect of the year was also highly significant. Application of P at 60 kg P_2_O_5_ ha^−1^ increased the HI by 29% and 16% during the first and second growing seasons, respectively. Similarly, the application of K increased the HI by 43% and 27% during 2020–2021 and 2021–2022 growing seasons, respectively.

**Figure 7:**
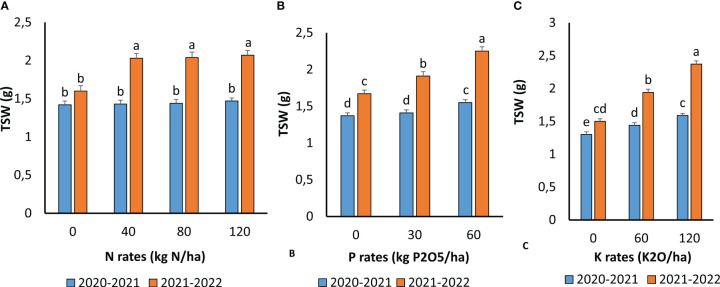
TSW as affected by N **(A)**, P **(B)** and K **(C)** application during the two cropping seasons 2020-2021 and 2021- 2022. Means followed by the same letters are not significantly different at p ≤0.05.

N, P, and K fertilization significantly enhanced the quinoa TSW. There was also an interaction between these three nutrients and the two growing seasons (**Figure 7**). N application did not significantly affect the TSW during the first growing season. However, the effect was significant during the second season, and 40 kg N ha^−1^ was optimal to increase TSW under the conditions of the present experiment. Although the environmental conditions of the two growing seasons were different, the same tendency of TSW increase following P and K application was observed. TSW increased with increasing P and K levels, and the application of 60 kg P_2_O_5_ ha^−1^ and 120 kg K_2_O ha^−1^ resulted in the highest values in both growing seasons.

### 3.7 N, P, and K uptake

The optimal quinoa nutrient requirements were considered as the minimum quantity at which optimal yield was attained. The crop N, P, and K uptake with response to N, P, and K application are presented in **Table 3**.

**Table 3:** Quinoa N uptake (a), P uptake (b), and K uptake (c) as affected by N, P, and K application during 2021–2022 growing season.

NPK (kg N–P_2_O5–K_2_O ha^−1^)	N uptake (kg N ha^−1^)		P uptake (kg P_2_O_5_ ha^−1^)		K uptake (kg K_2_O ha^−1^)	
0–0–0	26.3 ± 3.8	b	10.5 ± 1.7	a	86.6 ± 7.9	a
0–0–60	35.2 ± 12.8	b	13.5 ± 7.2	a	124.1 ± 55.4	a
0–0–120	41.0 ± 12.9	ab	17.9 ± 6.9	a	133.4 ± 23.7	a
0–30–0	33.5 ± 1.9	b	12.2 ± 1.7	a	110.6 ± 17.0 a	a
0–30–60	34.1 ± 2.5	b	16.2 ± 1.1	a	119.1 ± 6.5	a
0–30–120	42.3 ± 10.3	ab	17.9 ± 7.1	a	147.6 ± 54.37	a
0–60–0	44.0 ± 5.5	ab	17.9 ± 2.0	a	156.0 ± 31.0	a
0–60–60	51.7 ± 12.9	ab	24.7 ± 6.4	a	193.1 ± 41.3	a
0–60–120	60.3 ± 32.5	ab	14.6 ± 3.1	a	222.6 ± 115.0	a
40–0–0	40.5 ± 4.6	ab	11.9 ± 2.3	a	113.1 ± 6.4	a
40–0–60	42.3 ± 8.2	ab	14.4 ± 4.0	a	157.9 ± 68.9	a
40–0–120	55.6 ± 12.6	ab	14.4 ± 2.9	a	173.8 ± 31.6	a
40–30–0	42.6 ± 5.6	ab	16.4 ± 1.6	a	154.4 ± 19.0	a
40–30–60	42.8 ± 14.5	ab	15.9 ± 6.3	a	141.2 ± 21.2	a
40–30–120	62.0 ± 16.8	ab	19.8 ± 5.8	a	234.7 ± 53.5	a
40–60–0	40.3 ± 10.8	ab	14.1 ± 3.1	a	157.4 ± 43.8	a
40–60–60	72.2 ± 11.6	ab	22.4 ± 5.2	a	197.7 ± 49.4	a
40–60–120	65.2 ± 9.2	ab	27.6 ± 5.7	a	220.8 ± 18.2	a
80–0–0	54.9 ± 6.9	ab	14.3 ± 2.3	a	159.8 ± 5.7	a
80–0–60	57. ± 15.5	ab	13.7 ± 3.6	a	186.4 ± 37.4	a
80–0–120	65.5 ± 9.5	ab	16.4 ± 1.2	a	191.0 ± 7.2	a
80–30–0	76.5 ± 6.2	ab	23.3 ± 2.8	a	181.8 ± 12.1	a
80–30–60	55.4 ± 19.4	ab	19.3 ± 2.0	a	191.1 ± 60.6	a
80–30–120	52.0 ± 10.3	ab	21.4 ± 2.5	a	185.0 ± 33.2	a
80–60–0	46.5 ± 9.5	ab	14.2 ± 4.2	a	150.2 ± 11.2	a
80–60–60	51.4 ± 15.2	ab	20.1 ± 7.3	a	174.5 ± 49.0	a
80–60–120	70.6 ± 13.3	ab	26.0 ± 4.2	a	243.3 ± 31.3	a
120–0–0	56.6 ± 12.8	ab	13.5 ± 3.9	a	173.5 ± 11.2	a
120–0–60	62.0 ± 9.3	ab	18.5 ± 1.9	a	183.6 ± 27.4	a
120–0–120	71.6 ± 24.5	ab	20.1 ± 6.4	a	200.7 ± 82.0	a
120–30–0	56.7 ± 20.2	ab	16.5 ± 5.3	a	171.2 ± 25.5	a
120–30–60	57.1 ± 18.2	ab	17.4 ± 3.7	a	214.7 ± 40.2	a
120–30–120	96.2 ± 22.6	a	19.9 ± 3.6	a	328.1 ± 56.5	a
120–60–0	73.7 ± 9.5	ab	19.3 ± 1.6	a	192.2 ± 28.8	a
120–60–60	66.3 ± 19.3	ab	21.8 ± 4.8	a	225.0 ± 78.8	a
120–60–120	60.9 ± 24.0	ab	20.6 ± 7.3	a	206.3 ± 49.7	a

Data followed by the same letters are not significantly different at p ≤0.05.

We only considered quinoa nutrients uptake during the second growing season, where the temperature was not a limiting factor for growth and yield production. Results showed that 65.2 kg N ha^−1^ was the quinoa N uptake to produce optimal yield (1.1 t ha^−1^). The control treatment recorded the lowest nitrogen uptake, 26.3 kg N ha^−1^ (**Table 3**). Adding P at 30 kg P_2_O_5_ ha^−1^ and 60 kg ha^−1^ increased N uptake to the optimal level with lower N rates. This would be explained by the fact that P positively affected quinoa root system and enhanced N uptake from the soil. Total plant chemical analysis at physiological maturity showed that 27.6 kg P_2_O_5_ ha^−1^ was the optimal P uptake to attain the optimal grain yield (1.1 t ha^−1^) in this experiment. However, there was no significant difference between the P uptake of the different treatments. Compared to N and P, quinoa needed the K at high quantities. The optimal K uptake to attain optimal yield (1.1 t ha^−1^) was estimated to be approximately 220 kg ha^−1^.

### 3.8 Nutrient use efficiency

N use efficiency (NUE) was significantly affected by both N application and growing season (**Table 4**). The highest value was obtained with the application of 40 kg N ha^−1^. For both growing seasons, N rates higher than 40 kg N ha^−1^ decreased quinoa NUE. High difference between the growing seasons was also recorded, essentially due to high temperatures during the flowering and seed-filling stages that reduced the grain yield.

**Table 4:** Nutrient use efficiency during 2020–2021 and 2021–2022 growing seasons.

	Nutrient rates	2020–2021	2021–2022
**NUE (kg/kg N)**	0 kg N ha^−1^	0	0
	40 kg N ha^−1^	3.79 ± 0.59 Ba	22.54 ± 1.03 Aa
	80 kg N ha^−1^	2.18 ± 0.37 Bb	11.76 ± 0.66 Ab
	120 kg N ha^−1^	1.53 ± 0.26 Bb	8.16 ± 0.60 Ac
**PUE (kg/kg P_2_O_5_)**	0 kg P_2_O_5_ ha^−1^	0	0
	30 kg P_2_O_5_ ha^−1^	5.05 ± 0.69 Ba	29.47 ± 1.69 Aa
	60 kg P_2_O_5_ ha^−1^	3.49 ± 0.47 Bb	15.36 ± 0.82 Ab
**KUE (kg/kg K_2_O)**	0 kg K_2_O ha^−1^	0	0
	60 kg K_2_O ha^−1^	2.69 ± 0.35 Ba	14.52 ± 0.73 Aa
	120 kg K_2_O ha^−1^	1.89 ± 0.23 Bb	8.25 ± 0.45 Ab

For each nutrient use efficiency and rate, means followed by the same capital letters are not significantly different at p ≤0.05. For each nutrient use efficiency and growing season, means followed by the same small letters are not significantly different.

In the case of P, a significant difference was noticed between P application rates (**Table 4**). The effect of the growing season was also significant. However, a similar tendency was observed following P application during 2020–2021 and 2021–2022. The highest PUE values, 5.1 and 29.5, were recorded at 30 kg P_2_O_5_ ha^−1^ at the two growing seasons, respectively. In addition, the difference in PUE between the two growing seasons was highly significant and resulted in the highest values.

KUE was significantly affected by K application and growing season (**Table 4**). The application of K at 60 kg K_2_O ha^−1^ significantly enhanced the KUE, whereas higher rates (120 kg K_2_O ha^−1^) decreased this parameter. Similarly to NUE and PUE, lower values of KUE were recorded during the first growing season.

## 4 Discussion

Quinoa positively responded to macronutrient under semi-arid conditions in the present study. Among the three nutrients, N fertilization recorded the highest increase in plant height, total biomass, TSW, and grain yield. Similar responses were found in Pakistan by Imran et al. (2021) and Zahid et al. (2021), where N enhanced plant height and yield and its components. The role of N in promoting metabolic activity resulted in internode elongation and increased plant height with high N rates. N also has a structural role in the chlorophyll molecule. N fertilizer increased the leaf CCI, which enabled the plant to capture more sunlight energy by photosynthesis, enhancing plant growth and grain yield. Similar results were reported by various authors (Shams, 2012; Llaca Ninaja, 2014; Geren, 2015; Almadini et al., 2019).

Nevertheless, quinoa seems to be well adapted to poor soil conditions and is considered a crop with low input requirements. In this experiment, the optimal grain yield was obtained by 40 kg N ha^−1^. Calculating the C/N of the soil resulted in a value of 10, which characterizes an organic matter rapidly mineralized and released nutrients were available to the plant. We admit that the other part of quinoa N requirement was taken from soil organic matter decomposition and irrigation water. Thus, 40 kg N ha^−1^ was sufficient to attain optimal yield in this experiment (0.8 t ha^−1^). In addition, the application of nutrients in the 2020–2021 growing season might affect quinoa response in 2021–2022 growing season and enhanced the crop performance. The recommended fertilization is influenced by soil, water, and climatic conditions. Under drought and heat stress conditions of the Sahel, application of 25 kg N ha^−1^ with progressive drought resulted in the highest grain yield (Alvar-Beltrán et al., 2019). Wang et al. (2020) tested the response of quinoa to N rates under different water regimes, and they found that under rainfed conditions, N treatments from 37 to 50 kg ha^−1^ enhanced the grain yield and differed significantly from the control treatment, but not within themselves. Under the moderate temperate condition of Denmark, a slight increase in quinoa yield of 24.1% was achieved when the N supply increased from 40 to 160 kg N ha^−1^ and an increase of only 2.7% from 120 to 160 kg N ha^−1^ (Jacobsen et al., 1994). They suggest that the lack of response to increased N application could be relative to the high plantation density in the experimental site. Basra et al. (2014) compared the response of two quinoa cultivars to N rates from 0 to 125 kg ha^−1^ in a sandy loam soil with NPK levels of 0.042%, 11.52 mg kg^−1^, and 90 mg kg^−1^. Results showed that 75 kg N ha^−1^ gave the maximum grain yield of quinoa. Furthermore, Rivero (1985) showed that the highest yield was obtained with the variety Yanamarca at 120 kg N ha^−1^. These results were further confirmed by Leonardo (1985). Under Mediterranean climatic conditions of Turkey, N application at 150 kg ha^−1^ to a silty-clay loam soil, having an initial total N of 0.123%, was proved to be the best level for optimal grain yield (2.95 t ha^−1^), with 357% increase compared to the control (Geren, 2015). Similar results were obtained by Almadini et al. (2019) when evaluating the response of quinoa to N fertilization levels from 0 to 160 kg N ha^−1^. N at 160 kg ha^−1^ enhanced vegetative growth and increased yield by more than 750% compared to the control. Quinoa seemed to be well adapted to Pakistan’s environmental conditions (Akram et al., 2021). When quinoa was cultivated in heavy metals polluted soil using sewage water, a high grain yield (3.4 t ha^−1^) was recorded with the application of 75–50–50 of N–P_2_O_5_–K_2_O ha^−1^ (Ghous et al., 2022). Water availability and adequate temperature are two major environmental factors that control quinoa growth and yield (Taaime et al., 2022a). In our experiment, drought and high temperature were limiting factors for crop growth. Thus, quinoa’s potential yield was low, and quinoa did not respond to higher N application levels. This was observed during the first cropping season, where high temperatures occurring at the end of the growing cycle negatively affected the grain yield. As a result, the crop did not respond to different levels of N application. However, when irrigated with high salinity irrigation water in sandy soil, quinoa responded to higher levels of N up to 360 kg ha^−1^ (Shams, 2012). High N supply increased the N uptake and water root uptake and other nutrients essential for plant growth, which resulted in high yields (Ding et al., 2018). In addition, high N fertilization rates are recommended for high-potential varieties, and 300 kg N ha^−1^ was needed to produce 6–7 t ha^−1^ of grain yield (Gómez and Aguilar, 2016). In west-central Morocco (Agadir region), Hirich (2014) studied the combined effect of water stress and N supply. Results of this experiment showed that N application enhanced the grain yield under water stressed conditions, with the highest increase recorded at 240 kg N ha^−1^ under 25% of full water requirement. In our experiment, we believe that the positive effect of N on quinoa grain yield was attributed to its effect on TSW. N application increased the chlorophyll content, and more assimilates were directed to the grain formation. In another experiment, the same agronomic parameters were enhanced by higher N application rates (240 kg ha^−1^) with increased quinoa plant’s height, seed weight, and biomass (Fawy et al., 2017).

Generally, high N fertilization negatively affected the NUE. N rates higher than 40 kg N ha^−1^ decreased the NUE in the present experiment. Similar results were reported by Kakabouki et al. (2019), where N fertilization above 100 kg N ha^−1^ decreased the NUE of quinoa. However, our results showed that the NUE values were higher than other values found in the literature. Berti et al. (2000) found that 13.9 kg kg^−1^ was the highest NUE value reached with 75 kg N ha^−1^. In agreement with that, Almadini et al. (2019) reported an inverse relationship between N fertilization and NUE, with best value recorded at the 80 kg N ha^−1^. Related results were found by Shams (2012), where application of 90 kg N ha^−1^ corresponded to maximum NUE. This parameter also differs according to quinoa cultivars. Deza (2018) recorded that LM 89-77 genotype had the highest NUE with 46 kg of grain per kg of applied N. However, NUE in cereals averages 33%, indicating higher potential for improvement for quinoa crop (Quintero and Boschetti, 2009).

Based on total plant analysis at physiological maturity, it was found that quinoa absorbed 65.2 kg N ha^−1^ to produce 1.1 t ha^−1^ of grain yield and 4.1 t ha^−1^ of biomass. Schulte auf’m Erley et al. (2005) reported that quinoa absorbed 161.3 kg N ha^−1^ to produce 3.5 t ha^−1^ of grain yield.

K application also significantly affected the growth and yield of quinoa. Unlike N, quinoa responded to K up to the highest application rate (120 kg ha^−1^). Other work reported that quinoa responded to K application up to 120 kg ha^−1^ (Salim et al., 2019). The increase in the yield and its components recorded with high K rates could be attributed to the role the K plays in improving the weight and size of grain, which are important traits for quinoa quality (El-Sayed et al., 2023). This nutrient has been found to reduce the negative effect of drought by regulating root morphology and exudates and microbial community (Xu et al., 2021). However, grain yield decreased when K was applied at rates higher than 180 kg ha^−1^ (Salim et al., 2019). In addition, K application was proved to increase quinoa yield under normal and stressed saline conditions (Turcios et al., 2021). Our results showed that quinoa has a large requirement for K, with an uptake of 220 kg K ha^−1^ to produce 1.1 t of grain yield. Under irrigation in Burkina Faso, quinoa absorbed 42.8 kg K_2_O to produce 1 t of quinoa biomass (Alvar-Beltrán et al., 2021). A value that was similar to our results in this experiment, where quinoa needed 42.2 kg K_2_O to produce 1 t of total biomass. Under the conditions of the present experiment, KUE values were lower than those reported in the literature, where KUE reached 16.75 g g^−1^ (Rekaby et al., 2021).

Compared to N and K, the P was needed in lower quantities. Similar results were found by Alvar-Beltrán et al. (2021). They found that quinoa needs 3.7 kg of P_2_O_5_ to produce 1 t of quinoa biomass. In our experiment, quinoa responded positively to P application up to the highest level (60 kg P_2_O_5_ ha^−1^). In line with this, 88 kg P_2_O_5_ ha^−1^ was the optimum rate in sandy loam soil producing a 2.9 t ha^−1^ grain yield (Llaca Ninaja, 2014). In the present study, increasing P rates affected negatively the PUE. Our results were confirmed by Jorfi et al. (2022), where the highest PUE was obtained with the lowest P rate (50 kg P_2_O_5_ ha^−1^). However, the PUE values obtained in the present study were higher than other studies where quinoa PUE ranged from 5.9 to 14.9 g g^−1^(Lata-Tenesaca et al., 2021; Rekaby et al., 2021). Nutrients interaction was recorded for the plant height and CCI. The response of quinoa plant height to the highest rates of P and K decreased with increasing N levels, with an increase over the control of 31%, 20%, and 16% at 40, 80, and 120 kg N ha^−1^. Thus, the combination of N, P, and K for optimal plant height was 80 kg N ha^−1^, 60 kg P_2_O_5_ ha^−1^, and 120 kg K_2_O ha^−1^. Unlike the plant height, no significant effect of K was recorded for the CCI at the highest N and P rates. However, the K significantly affected the CCI when P was not applied. This may be attributed to the fact that at high levels of N and P, the plant developed deep root system able to explore the nutrients necessary for the CCI synthesis, and no significant effect was recorded with the addition of the K fertilizer.

In Peru, N, P, and K fertilization enhanced plant height, panicle length, number of branches, and quinoa yield (Chavez Melgarejo, 2018). When testing the response of quinoa to the application of different N, P, and K rates, it was found that 160 kg ha^−1^ of N, 100 kg ha^−1^ of P_2_O_5_, and 160 kg ha^−1^of K_2_O resulted in the highest grain yield (6.6 t ha^−1^) (Chavez Melgarejo, 2018). In another study, applying fertilizers at 80 kg N ha^−1^, 80 kg P_2_O_5_ ha^−1^, and 90 kg K_2_O ha^−1^ resulted in the highest yield, 4.3 t ha^−1^, with an increase of 141% compared to the control.

From the literature discussed, it was concluded that quinoa response to fertilization varied according to cultivars, soil types, climatic conditions, and agronomic management practices. This highlights the importance of evaluating quinoa nutrient requirements under different environmental conditions to develop adequate recommendations for the sustainable cultivation of quinoa and increasing agronomic productivity in arid regions.

## 5 Conclusion

The finding of our experiment showed that quinoa responded positively to N, P, and K applications under semi-arid conditions in Morocco. The optimal fertilizer combination was 40–60–120 kg ha^−1^ of N–P_2_O_5_–K_2_O and resulted in high plant height (81.6 cm), CCI (46.41), and grain yield (0.8 t ha^−1^). Our results showed that N rates higher than 40 kg ha^−1^ did not significantly enhance quinoa grain yield. However, this parameter continued to increase with higher P rates up to 60 kg P_2_O_5_ ha^−1^ and K rates up to 120 kg K_2_O ha^−1^. Thus, further experiments should be conducted to evaluate quinoa response to P and K rates higher than those tested in our experiment. Regarding quinoa nutrients uptake, quinoa needed 60 kg N, 26 kg P_2_O_5_, and 205 kg K_2_O to produce 1 t of grain yield.

## Data availability statement

The raw data supporting the conclusions of this article will be made available by the authors, without undue reservation.

## Author contributions

NT, ME, and KE contributed to the conception and design of the study. NT organized the database and performed the statistical analysis. NT wrote the first draft of the manuscript. RB, AO, KE, ME, and RC-A contributed to the manuscript revision and correction. All authors contributed to the article and approved the submitted version.
